# Rejuvenating Strategies of Tissue-specific Stem Cells for Healthy Aging

**DOI:** 10.14336/AD.2018.1119

**Published:** 2019-08-01

**Authors:** Min-jun Wang, Jiajia Chen, Fei Chen, Qinggui Liu, Yu Sun, Chen Yan, Tao Yang, Yiwen Bao, Yi-Ping Hu

**Affiliations:** ^1^Department of Cell Biology, Center for Stem Cell and Medicine, Second Military Medical University, Shanghai 200433, China; ^2^Department of Diagnostic radiology, University of Hong Kong, Hong Kong 999077, China

**Keywords:** Rejuvenation, Stem cell aging, Tissue homeostasis, Regenerative impairment, Stem cell niche, Systemic environment

## Abstract

Although aging is a physiological process, it has raised interest in the science of aging and rejuvenation because of the increasing burden on the rapidly aging global population. With advanced age, there is a decline in homeostatic maintenance and regenerative responsiveness to the injury of various tissues, thereby contributing to the incidence of age-related diseases. The primary cause of the functional declines that occur along with aging is considered to be the exhaustion of stem cell functions in their corresponding tissues. Age-related changes in the systemic environment, the niche, and stem cells contribute to this loss. Thus, the reversal of stem cell aging at the cellular level might lead to the rejuvenation of the animal at an organismic level and the prevention of aging, which would be critical for developing new therapies for age-related dysfunction and diseases. Here, we will explore the effects of aging on stem cells in different tissues. The focus of this discussion is on pro-youth interventions that target intrinsic stem cell properties, environmental niche component, systemic factors, and senescent cellular clearance, which are promising for developing strategies related to the reversal of aged stem cell function and optimizing tissue repair processes.

Tissue-specific stem cells, located in differentiated tissues, are imbued with a self-renewal potential and the differentiated capacity to generate multiple cell types within a tissue. In a common physiological event and injury response, the resident stem cells are able to perform asymmetric divisions to generate daughter cells that self-renew to preserve stem cell identity or commit to differentiation, thereby contributing to tissue homeostasis and repair. The regenerative roles in stem cell populations vary according to their host tissues. For example, neural stem cells (NSCs) are important for the generation of new neurons in the brain; however, they play a limited role in damage repair. In contrast, skeletal muscle stem cells (MuSCs) play a minimal role in muscle maintenance, whereas they vigorously engage in regeneration after injury. Hematopoietic stem cells (HSCs) and intestinal stem cells (ISCs) perform both functions, contributing to the ongoing production of differentiated cells and tissue injury repair [1, 2]. However, these stem cells in many tissues have been found to undergo profound changes with age, exhibiting a blunted responsiveness to tissue injury, dysregulation of proliferative activities and declining functional capacities. Moreover, the impairment of stem cell function with age results in the gradual loss of tissue homeostasis and injured tissue regeneration, which translates into dysfunction in aged organisms such as muscle weakness, osteoporosis, cognitive disorders, graying and loss of hair [3]. Although the mechanistic basis for age-associated stem cell decline is not completely understood, numerous studies have shown that stem cell aging is mediated by cell autonomous factors, such as the accrual of DNA damage, epigenetic dysregulation, loss of polarity, or disruption of signaling pathways, or extrinsic factors, including the stem cell niche and systemic environment that provides signals via paracrine or juxtacrin [3-6]. From a clinical perspective, it raises the consideration that the underlying mechanisms of the stem cell aging process may be pharmacologically intervened. In this paper, we have summarized the characteristics of aged tissue-specific stem cells and their regulated effects on different tissues and organs. Importantly, we focus on demonstrating increasing number of promisingly rejuvenating interventions of aged tissue stem cells by targeting their intrinsic mechanisms, extrinsic environment, or clearance of senescent cells. We also discuss the potential regenerative medicine strategies to restore age-related changes of stem cells, which would hopefully enhance the homeostasis and repair capacity of old and diseased tissues.

**Figure 1.**
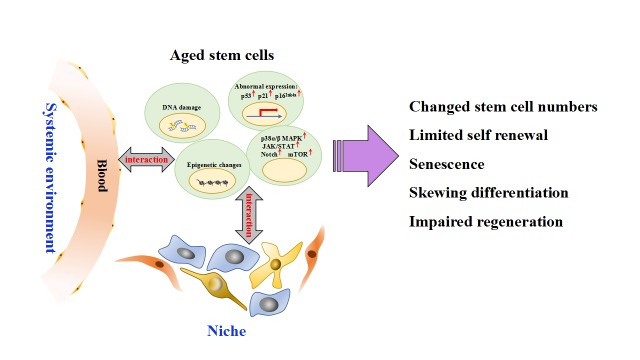
Summary of the underlying mechanisms contributing to the age-related changes in tissue-specific stem cells During aging, stem cells are controlled by intrinsic effectors including DNA damage accumulation, epigenetic changes, abnormal genes expression, and dysregulated cell signaling pathways, as well controlled by extrinsic mechanisms that are consist of stem cell niche and systemic environment. With these intrinsic effectors and cell-extrinsic regulations, the aged stem cells display numbers changes, limited self-renewal, senescence, skewing differentiation, and impaired regeneration.

## Aging in tissue-specific stem cells

Over the past decade, it has become evident that stem cells in various tissues undergo aging-associated changes, which are critical for the decline of tissue homeostasis and repair. In general, the hallmarks of tissue stem cell aging contain altered available stem cells, the loss of self-renewal, a disrupted differentiated capacity, increased apoptosis, and senescence. For example, the numbers of HSCs and ISCs increase several-fold with age; however, their functions decrease compared to their youthful counterparts [1, 7, 8]. In contrast, a reduction in stem cell numbers has been observed in skeletal muscle stem cells, neural stem cells, melanocyte stem cells and germline stem cells [9-12]. The loss of balance between stem cell self-renewal and differentiation is typically observed. Aged HSCs show alterations in the distribution of the cell polarity, which results in a symmetric division to generate two differential daughter cells for cell replacement while not preserving the stem cell identity [13, 14]. Consistent with serial transplantation, the self-renewal capacity of aged HSCs is substantially less than those isolated from young donor mice [15]. Defects in cell polarity are also observed in aged MuSCs, which disturb the asymmetric division, leading to the increased generation of committed progenitors and a reduction in self-renewal [16, 17]. Similarly, melanocyte stem cells of hair follicles in elderly individuals tend to differentiate into melanocytes, which account for gray hair and stem cell loss [18].

Another typical age-dependent phenotype of tissue stem cells is the bias in generating differentiated cell types. For example, aged HSCs are more likely to differentiate towards the myeloid lineage at the expense of the lymphoid lineage [19]. This skewed differentiation potential of aged HSCs is consistent with the incidence of myeloid malignancies in aged individuals [20]. Satellite cells during aging tend to differentiate towards a fibrogenic lineage rather than a myogenic lineage, largely dysregulated by Wnt and transforming growth factor-β (TGF-β) signaling [21, 22]. Furthermore, aged cycling NSCs exhibit an astrocytic bias, which is consistent with a failure to produce newborn neurons in the aged brain [23]. Senescence is also widely found in aged adult stem cells and contributes to impairing the regenerative capacity of a tissue by limiting stem cell function in a cell-autonomous way. At geriatric ages (28 months or more in the mouse), quiescent MuSCs transit to a pre-senescent state, with the consequent upregulation of the cell-cycle inhibitor p16^INK4a^ [24, 25]. A similar quiescence-to-senescence transition, associated with increased levels of p16^INK4a^, is observed in aged HSCs.

Stem cell aging is driven not only by stem cell intrinsic factors but also the aging niche and systemic environment (Fig. 1). For example, telomere shortening with a lack of sufficient telomerase activity is a primary molecular cause of aging. A short telomere limits the self-renewal and the proliferative capacity of HSCs, disrupts neuronal differentiation and neuritogenesis, as well as exhibits stem cell depletion [26]. Telomere shortening results in DNA damage accumulation, which alters gene function through mutations or chromosomal rearrangements. This change will lead to aberrated expressions and activities in genes that control stem cell differentiation and self-renewal [27, 28] Other cell intrinsic players of aging in stem cells included mitochondrial dysfunction, abnormal regulation of p38 mitogen-activated protein kinase (MAPK), Notch/ c-Jun N-terminal kinase (JNK), bone morphogenetic proteins (BMPs), and insulin like growth factor (IGF) signaling, which explain, in part, the loss of stem cell self-renewal, decreased proliferation and inappropriate differentiation [16, 29-33]. Stem cells reside in a specialized microenvironment called a niche. Age-dependent alterations in the components of the stem cell niche coupled with abnormal expression of cytokines and extracellular matrix protein secreted by niche cells have been reported to influence all aspects of stem cell function: quiescence, proliferation, multi-potency, and differentiation [6, 34]. Heterochronic transplantation and heterochronic parabiosis demonstrate that the perturbation of systemic soluble molecules secreted by tissues in the body may directly or indirectly influence stem cell proliferation and the regeneration potential [35-37]. These soluble molecules can be hormones, growth factors, and any other signaling molecules or immune-derived signals secreted by infiltrating immune cells.

Stem cells are one of the longest-lived cells in an organism; thus, they play a vital role in organismal aging and are highly correlated with the occurrence of disease. HSC aging with skewed differentiation contributes to an increase of acute myeloid leukemia with age. Less lymphocyte production of aged HSCs also manifests as a decreased immune response, suffering from the increased susceptibility of the elderly to infectious diseases, autoimmunity, anemia, and cancers [38]. The dysfunction of MuSCs in aged individuals is characterized by a loss of the balance between quiescence, self-renewal and differentiation, which results in progressive muscle mass decline, muscle strength weakness, and regenerative failure, generally referred to as “sarcopenia” [39]. Age-dependent changes of NSCs notably contribute to cognitive decline with learning and memory deficits, movement disorders, forgetfulness, and distractibility in the elderly, which might underline several diseases, including stroke, Alzheimer’s and Parkinson’s diseases [40]. The incidence of age-related neurodegenerative disorders is expected to increase, given that the number of adults over the age of 65 years is expected to increase to 20% of the US population by the year 2050 [41]. In addition, the aging of human ISCs contributes to the increase in colorectal cancer incidence with age [42]. Melanocyte stem cells with a loss of function might be the root cause of age-related hair graying [43].

### Intrinsic rejuvenating strategies of aged stem cells

#### Reversing DNA damage in aging stem cells

DNA damage accumulation is critical for age-dependent loss of tissue-specific stem cell function. This type of accumulation is dependent on the attenuation of the DNA repair and response pathway. For example, DNA damage markers, such as histone H2A phosphorylation and comet tails, have been quantified in HSCs and MuSCs from young and old mice and indicated strand breaks significantly accrue in HSCs and MuSCs during aging [35, 44, 45]. Accumulation of DNA damage in aged stem cells indicates a decrease in the function of appropriate repair mechanisms [46]. Transcriptome analysis of aged HFSCs reveals that the sustained DNA damage response in cycling cells during aging leads to a loss of the stem cell population from the skin through terminal epidermal differentiation [47]. It is therefore reasonable to suggest that an increase in the activity of DNA repair pathways may slow down or prevent the accumulation of age-related defects in stem cells and thereby promote the healthy function of aged tissues. For instance, deletion of the DNA damage sensor Atm causes an elevation of reactive oxygen species (ROS), loss of HSC quiescence, defects in repopulating capacity, and ultimately a depletion of the HSC pool [48]. But antioxidant treatment in Atm^-/-^ HSCs will rescue the reduced repopulating potential after serial transplantation. Genetic disruption of DNA repair pathways in mice further accelerates premature aging phenotypes [49]. Moreover, transgenic mice overexpressing Sirt6 display an extended lifespan via altering the IGF1 signaling pathway, which is a key factor in the regulation of the lifespan [50]. In a recent report, Piwi overexpression of ISCs is sufficient to allay their age-related retrotransposon expression, DNA damage, apoptosis, and mis-differentiation phenotypes, thereby improving epithelial homeostasis [51].

Telomeres are considered to cap chromosome ends thus preventing an activation of DNA damage responses and the evolution of chromosomal instability [52]. However, a gradual decline of the telomere length that occurs through the loss of telomerase during aging has been observed in mouse and human tissues. In the mouse model, the loss of telomerase displays telomere shortening, stem cell depletion, and impaired tissue injury responses. However, with telomerase reactivation, telomerase reverse transcriptase (TERT)-deficient mice extend telomeres and reverse degenerative phenotypes. Protection of telomeres 1A (Pot1a), a component of the Shelterin complex that protects telomeres, is highly expressed in young HSCs, whereas it progressively declines with age. In aged mice, treatment with exogenous Pot1a protein could reverse the HSC activity and sustain their self-renewal [53]. Notably, neural stem cells from telomerase RNA component mTERC-reactivated animals also restore their proliferative capacity and recover neurogenic function [54].

#### Repressing cell cycle inhibitor levels in aged stem cells

Increased expression of several cell cycle inhibitors, such as p53/p21, p16^Ink4α^, p19^Arf^, and p57^Kip2^ can lead to an essentially irreversible arrest of cell division and promote stem cell senescence. In MuSCs, HSCs, and NSCs, the expression of p16^Ink4α^ accumulates with age, but p16^Ink4α^ repression through various methods can improve the function of aged stem cells and prevent cellular senescence [55-58]. For example, silencing of p16^Ink4α^ expression in geriatric satellite cells restores their quiescence and regenerative potential [58]. In a potentially insightful study, Garcia-Prat and his colleagues showed that autophagy is critical to the prevention of stem cell senescence by repressing the expression of p16^Ink4α^, and treatment with pharmacological rapamycin to stimulate autophagy could rejuvenate the MuSCs [59]. Old p16^Ink4α-/-^ HSCs also exhibit an increased cell cycle activity and an enhance engraftment capacity after transplantation [55]. Deletion of the Ink4α locus results in increased proliferation and neurogenesis from neural progenitors in the subventricular zone (SVZ) compared to young counterparts, but not in the subgranular zone (SGZ) [56].

#### Intervening in epigenomic modification process to rejuvenate stem cell function

Epigenetic regulation including DNA methylation and histone modifications, enables cells to possess the same genetic sequence but carry out different functions. Many studies point that altered epigenetic marks of aging stem cells not only alter the transcriptional programs that dictate the function of the stem cells but also alter the potential differentiation towards distinct effector lineages [60, 61]. To support this, aged satellite cells display a global increase in the repressive trimethylation of lysine 4 on the histone H3 protein subunit (H3K27me3) mark [62]. Recently, aberrant global and site-specific induction of active chromatin marks such as Hoxa9, has been investigated in aged satellite cells, while the inhibition or deletion of homeobox A9 (Hoxa9) can improve MuSC function and muscle regeneration in aged mice [63]. Similar studies indicate that the activated H3K4me3 mark in aged HSCs increases the genes involved in maintaining HSC identity, while significantly repressing differentiation-promoting genes [64]. Depending on an altered epigenome and transcription, reprograming aged HSCs into induced pluripotent stem cells (iPSCs) and then redifferentiating these cells into HSCs indicated that the HSC-aging state could be reversed [65]. Another example of successful rejuvenation comes from the study, in which aged HSCs express a lower level of the chromatin organizer Satb1 than their young counterparts, while overexpression of Satb1 can improve their ability to generate lymphoid progeny via epigenetic reprogramming [66]. DNA demethylation can also occur via progressive oxidation of methylcytosine (mC) catalysis by ten-eleven translocation (Tet) family enzymes. For example, Tet2 catalyzes the oxidation of 5-methylcytosine (5mC) to 5-hydroxymethylcytosine (5hmC), a potential epigenetic regulator of aging. An increase of Tet2 in the hippocampus of aged animals can raise 5hmC production, which restores adult neurogenesis to youthful levels and enhances cognitive function [67].

Another epigenetic regulation is mediated by histone modifications. Acetylation of histone tails alters the charge of the histone, thereby loosening compacted chromatin and enabling a more open and permissive transcriptional state. Interestingly, it is reported that aged HSCs show decreased levels and altered cellular distribution of histone H4 lysine 16 acetylation (H4K16ac), in contrast to young HSCs with high levels of polarized H4K16ac expression. However, the altered H4K16ac in these aged HSCs is reversed by pharmacological inhibition of cell division cycle 42 (Cdc42) activity, accompanied by the restoration of HSC function [13]. Sirtuin family comprises mitochondrial histone deacetylases. In particular, although Sirt3 and Sirt7 have been found to be decreased in aged HSCs, overexpression of Sirt3 or Sirt7 can rescue aging-associated HSC functional defects, including increasing the HSC reconstitution capacity and reducing the aged HSC myeloid bias [68, 69].

**Table 1:** Rejuvenation of tissue-specific stem cells via therapeutic molecules on their niche.

Intervening approach	Target cell	Mechanism	Rejuvenation on function	References
Fgfr1 inhibitor SU5402 or Spry1 overexpression	MuSCs	reducing FGF signaling	loss of quiescence, regenerative capacity	[24]
Fibronection injection	MuSCs	rescue FAK signaling	proliferative and myogenic potential	[78]
TS2/16	MuSCs	activation of β1-integrin/FGFR	regenerative capacity	[17]
Tyr AG 490	MuSCs	inhibition of JAK/STAT	satellite cell number; self-renewal; regenerative capacity	[70]
5,15 diphenylporphrine	MuSCs	inhibition of JAK/STAT	satellite cell number; self-renewal; regenerative capacity	[70]
Sodium salicylate	MuSCs	inhibition of NF-κB signaling	regenerative capacity	[79]
SB-505124	NSCs	blockade of TGFβ signaling	proliferation of stem cells; neurogenesis	[82]
Lateral ventricle choroid plexus (LVCP) secretome	NSCs	unknown	proliferation, self-renewal, and differentiation	[32]
Loss of Dkk1	NSCs	increase of Wnt activity	self-renewal; number of neuronal progenitors; neurogenesis	[83]
Rantes knockout	HSCs	decreased mTOR activity	myeloid skewing; engraftment potential	[80]
Inactivation of the gene encoding Fbxw7	HSCs	activation of Notch signaling	HSCs numbers	[81]

#### Restoring aged stem cell function by targeting cell signaling pathways

Signals can directly influence all aspects of stem cell functions including quiescence, proliferation, and differentiation. Signaling pathways involving p38-MAPK, janus kinase (JAK)/ signal transducers and activators of transcription (STAT), Notch, and mechanistic target of rapamycin kinase (mTOR) contribute to the modulation of tissue stem cell functions, and their changes with age could affect tissue maintenance and repair systems. Hence, the proper modulation of these pathways is related to the reverse senescence of adult stem cells, which present the enhanced regenerative capacity of the tissues.

For example, following overactivation of the p38α/β MAPK pathway, aged satellite cells are over-activated, and then increasingly generate their committed progenitors, while reducing self-renewal [16]. However, pharmacological inhibition of p38α/β MAPK in aged satellite cells is able to restore the engraftment potential and improve their self-renewal ability by restoring asymmetric division [16, 29]. JAK/STAT is a cytokine receptor pathway that increases with age, but its inhibition has been shown to restore muscle stem cell expansion [70, 71]. In contrast, Delta/Notch signaling decreases with age, leading to the decreased activity of skeletal muscle stem cells and impairment of aged muscle regeneration. Once stimulation of Notch signaling, the ability of satellite cells in proliferation and regeneration will be restored [72, 73]. Compared to young mice, the activity of mTOR signaling in HSCs from old mice is reported to increase. While treatment with the mTOR inhibitor rapamycin is found to reverse the aging-associated increase in HSC numbers, restore reconstitution potential and self-renewal activity [74]. These effects are conserved in MuSCs and other epithelium stem cells, which indicates that age-dependent stem cell loss in the trachea and muscle can be prevented by limiting mTOR activity [75].

**Table 2:** Intervention in systemic environment to rejuvenate function of tissue-specific stem cells.

Intervening approach	Target cell	Mechanism	Rejuvenation on function	References
Frizzled-related protein 3 (sFRP3) incubation	MuSCs	suppression of Wnt signaling	proliferative potential; muscle regeneration	[22]
Dickkopf-1 (Dkk1) injection	MuSCs	suppression of Wnt signaling	muscle regeneration	[22]
TGF-beta receptor kinase inhibitor	MuSCs	attenuating TGFβ signlling	regenerative potential	[21]
Recombinant GDF11 injection	MuSCs	unknown	regenerative potential	[35]
Oxytocin	MuSCs	activation of MAPK/ERK signaling	MuSC activation and proliferation; regenerative potential	[91]
Recombinant GDF11 injection	NSCs	activation of TGFβ signaling	self-renewal; differentiation potential; neurogenesis	[36]
GnRH I injection	NSCs	unknown	neuronesis; cognitive function	[87]
CCL11-specific neutralizing antibody	NSCs	unknown	neuronesis; cognitive function	[37]
N-acetylcysteine incubation	MSCs	Scavenging reactive oxygen species (ROS)	aging phenotypes	[85]
4-hydroxytamoxifen (4-OHT) injection	Skin	blockade of NF-κB	age-associated gene expression; proliferation	[86]
Recombinant GDF11 injection	Renal Epithelial cell	Upregulating ERK1/2 pathway	proliferative capacity; renal repair	[93]

### Extrinsic rejuvenating strategies of tissue-specific stem cells

#### Reversing stem cell niche

It is known that tissue-specific stem cells are located in niches. The niche components can be considered somatic and stromal cells, immune cells, extracellular matrix (ECM), innervating neuronal fibers, and the vasculature. Although the niche structure varies among the different adult stem cell types, the stem cell niche provides essential cues to influence cell fate decisions [76, 77]. The aging of niche cells and age-dependent alterations in the components of stem cell niche are able to cause a loss stem cell function. Thus, modifications of the stem cell environment can rejuvenate the function of aged stem cells (Table 1).

Fibroblast growth factor-2 (FGF-2), for example, is upregulated in the aged satellite cell microenvironment, whereas inhibition of FGF signaling can rescue the self-renewal capacity of old MuSCs [24]. In addition, the cell surface receptor β1-integrin and the ECM protein fibronectin are dysregulated in aged MuSCs, and reconstitution of these components is able to restore the muscle regenerative capacity [17, 78]. Niche-derived nuclear factor-_ k_B (NF-_k_B) signaling increases with aging and impairs satellite cell function; however, administration of an NF-_k_B inhibitor can restore the lost function of MuSCs [79]. Rante/ C-C motif chemokine ligand 5 (Ccl5) cytokine, which is highly expressed in the local niche and aged blood, contributes to the age-associated myeloid skewing. While knockout of Rantes can increase lymphoid lineages and improve the engraftment potential after transplantation [80]. Notably, the vasculature is an important component of the HSC niche, and niche-forming vessels are reduced during aging. However, the vessels can be restored by activation of endothelial Notch signaling [81]. Similarly, blockade of TGF-β signaling with neutralizing antibody or the pharmacological TGF-β inhibitor have been found to recover the neurogenesis of aged mice [82]. A recent study identifies the lateral ventricle choroid plexus (LVCP), a primary producer of cerebrospinal fluid (CSF), as an important niche component for NSCs affected by aging [32]. Reduced secreted molecules of LVCP, such as BMP5 and IGF-1, can improve the function of old NSCs. Moreover, by inducible loss of dickkopf WNT Signaling Pathway Inhibitor 1 (Dkk1) at the expense of increasing their Wnt activity significantly increases the self-renewal of neural stem cells and improves spatial learning and memory of the older mice [83].

#### Systemic environment to reverse stem cell aging

In addition to stem cell niche, aging also causes changes in circulating signals that directly or indirectly impact functions of tissue stem cells (Table 2). These signals include soluble molecules secreted by any tissue in the body, which can be hormones, growth factors, and other signaling molecules or immune-derived signals secreted by infiltrating immune cells. Wnt ligand level is higher in old mouse serum and canonical Wnt signaling directly antagonizes Notch signaling in satellite cells [22, 84]. But Wnt inhibitors effectively restored the satellite cell function in aging [22], and a similar result is obtained in aged mesenchymal stem cells [85]. Consistent with the transgenic modified model, the skin can rapidly revert to a more youthful state and manifest the molecular signature of youthfulness when treated with an NF-_k_B inhibitor [86]. The level of TGF-β is significantly increased in old human and mouse serum, which causes the damage and senescence of satellite cells. However, blockage of TGF-β signaling can reverse the activity of satellite stem cells, improving the myogenesis of aged mice [21]. In addition, systemic treatment with gonadotropin releasing hormone I (GnRH I) in aged mice is found to increase neurogenesis and improve cognitive function [87]. Elevated levels of chemokine CC-chemokine ligand 11 (CCL11) is the reason for learning and memory deficits in aged mice, and the function can be rescued with the injection of antibodies that neutralize CCL11 [37].

Heterochronic parabiosis shows that the exposure of old skeletal muscle to a youthful systemic environment can promote efficient satellite cell activation [72]. Similar results with enhanced neurogenesis and reversed cardiac hypertrophy are also found in the aging nervous system and heart [88]. Furthermore, the injection of plasma from young mice into the circulation of aged mice has recently been shown to induce a more youthful state of cells in the brain, muscle, and liver of old animals [88-90]. Intriguingly, circulating growth differentiation factor 11 (GDF11) and oxytocin are considered to have a ‘rejuvenating’ effect. The treatment of aged mice with recombinant GDF11 or oxytocin reverses the dysfunction of aged satellite cells and restores robust regenerative function [35, 91]. GDF11 supplementation in old mice further reverses hypertrophy in cardiac muscle [92], enhances neurogenesis in the brain, restores tubular regeneration in kidney injury [93], and improves physical activity [36]. However, Egerman et al claimed that there is an increased GDF11 level in aged rats and humans, which significantly inhibits muscle regeneration and decreases muscle stem cell expansion [94]. Associated with this opposite data, the effects of GDF11 on skeletal muscle and its systemic changes with aging have recently been controversial [94-97], indicating the need for further studies to fully determine whether this promising multi-target factor will be useful in therapies aimed at enhancing the regenerative capacity.

#### Rejuvenating strategy by clearing senescent cells and decreasing SASP

Senescent cells accumulate with aging in several tissues of humans and animals, which is a common feature of age-related pathologies [98-101]. Not only differentiated cells but also tissue-specific stem cells become senescence during aging. Moreover, the complex senescence-associated secretory phenotype (SASP) is highly expressed with accumulated senescent cells, which can alter the microenvironment and contribute to age-related pathologies. For example, the tissue regenerative capacity is impaired by the limited stem cells function because of their senescent state. And this decreased regenerative capacity is also regulated by the SASP that is secreted by senescent cells [102].

Thus, the selective clearance of senescent cells and SASP suppression will be a promising therapy for age-related diseases. This concept has been successfully tested in progeroid and physiologically aged mouse models. It is reported that genetically induced senescent cells clearance can attenuate age-related deterioration of several organs, without apparent side effects [103, 104]. Clearance of senescent intimal foam cells attenuates the pathologies of atherosclerosis at all stages of pathogenesis [105]. In addition, pharmacological interventions, such as the senolytic agent ABT263, have been reported to effectively deplete senescent HSCs and MuSCs when orally administered to aged mice [106]. ABT263 induces apoptosis of senescent cells by inhibiting the expression levels of the anti-apoptotic proteins B-cell lymphoma 2 (BCL-2) and B-cell lymphoma xL (BCL-xL). The selectively kill of senescent cells is sufficient to improve the function of the remaining healthy population of HSCs and MuSCs, which suggests that putative senescent cells in the bone marrow and muscle may secrete factors that negatively affect the HSC and MSC potential. A recently reported study also demonstrates that accumulation of senescent cells promotes hepatic fat accumulation and steatosis, while the elimination of senescent hepatocytes by gene-meditated ablation or treatment with senolytic drugs is able to reduce overall hepatic steatosis [107]. Senolytic treatment with Dasatinib and Quercetin results in significant reductions in senescent cells of aged mice and significantly improves vasomotor function [108].

It is also reported that fat cell progenitors, preadipocytes, become senescent in adipose tissue with aging and acquire a senescence-associated secretory phenotype, thus contributing to adipose tissue inflammation. But JAK inhibitors can reduce the expression of SASP in senescent preadipocytes and alleviate age-related adipose tissue and systemic inflammation [109]. Rapamycin can selectively blunt the pro-inflammatory phenotype of senescent cells, diminish NF-ҝB transcriptional activity, and ameliorate age-related pathologies [110]. Recent study shows that the clearance of senescent cells with UBX0101 treatment slows the development of naturally occurring osteoarthritis (OA) and post-traumatic OA in aged mice. Furthermore, selective removal of senescent cells also decreases the expression of inflammatory markers [111].

### Conclusions

Improving the health span of elderly individuals takes on an increasing urgency as the human lifespan continues to increase, and even small gains in the health span can substantially lessen the impact of an aging population on the health care system and the economy. As stem cells are the longest-living cells within an organism, stem cell aging is highly relevant as a driver of organismal aging, health and longevity. In this review, we demonstrate that by targeting aging mechanisms, the aging associated phenotypes and functions of tissue-specific stem cells can be reversed. These restorative interventions hold promise for the possibilities of regenerative medicine and the treatment of many age-related diseases and dysfunctions, including sarcopenia, heart failure, deficient immune function and neurodegeneration. For example, sarcopenia is considered as a result of MuSC-associated loss of muscle regeneration. This deficit can be overcome by pharmacological inhibitors that target micro-environmental factors via the Wnt, bFGF, and Notch pathways. Skewing differentiation of aged HSCs underlying the decreased production of B- and T-lymphocytes is the major reason for the immunity deficit. To date, the reported rational interventions for reversing skewing differentiation target both intrinsic and extrinsic mechanisms via pharmacological inhibition of the cdc42 activity and inflammatory cytokine Rates neutralizing antibody. Neurodegeneration has been correlated with a decline in both the number of NSCs and the proliferative expansion of progenitor cells. Intrinsic perturbations via repressing p16 expression and extrinsic interventions by administering a pharmacological TGFβ inhibitor or CCL11 neutralizing antibody might be promising strategies for restoring neurogenesis. Nevertheless, how does aging research in animal models relate to humans? Further studies should focus on translating the successful rejuvenating regimes into clinical therapies of aged-associated diseases.
